# BMC *Pulmonary Medicine* reviewer acknowledgement 2015

**DOI:** 10.1186/s12890-016-0196-2

**Published:** 2016-02-26

**Authors:** Dirk Krüger

**Affiliations:** BioMed Central, Floor 6, 236 Gray’s Inn Road, London, WC1X 8HB UK

## Abstract

The editors of BMC Pulmonary Medicine would like to thank all our reviewers who have contributed to the journal in Volume 15 (2015).

**Alen Faiz**

Netherlands

**As Hoza**

Tanzania

**Adam Collison**

Australia

**Adrian Ceccato**

Argentina

**Afroditi Boutou**

Greece

**Michelle Murray**

Ireland

**Aisling McGowan**

Ireland

**Akash Verma**

Singapore

**Nobuhiro Akuzawa**

Japan

**Albert Lim**

Singapore

**Anantham Devanand**

Singapore

**Andras Bikov**

Hungary

**Andrea Wolfler**

Italy

**Andreas Hector**

Germany

**Andrea Zanoni**

Italy

**Andrew Argent**

UK

**Anjali Ralhan**

Germany

**Anna Selvaggio**

USA

**Annemarie Lee**

Australia

**Ozsancak Ugurlu**

UK

**Luis Nannini**

Argentina

**Carlos Nigro**

Argentina

**Anand Agrawal**

India

**Kjetil Ask**

Canada

**Joerg Mattes**

Australia

**Paul Thomas**

Australia

**Narelle Cox**

Australia

**Uwana Evers**

Australia

**Chris Del Mar**

Australia

**Maher Almatar**

Australia

**Rakesh Kumar**

Australia

**Dino Bee Aik Tan**

Australia

**Peter Franklin**

Australia

**Jodie Simpson**

Australia

**Bircan Erbas**

Australia

**Dianne Goeman**

Australia

**Richard Kim**

Australia

**Vanessa McDonald**

Australia

**Andre Schultz**

Australia

**Biarta Rhys-Jones**

Australia

**Graeme Zosky**

Australia

**Christopher Zappala**

Australia

**Mark Everard**

Australia

**Leanne Rodwell**

Australia

**Vicki Clifton**

Australia

**Vanessa Murphy**

Australia

**Dennis Lau**

Australia

**Bronwyn Berthon**

Australia

**Paul Robinson**

Australia

**Rosalba Courtney**

Australia

**Gavin Pinniger**

Australia

**Jutta Horejs-Hoeck**

Austria

**Balazs Hegedus**

Austria

**Antoni Xaubet**

Spain

**Sandra Baldacci**

Italy

**Begum Ergan**

Italy

**Benoit Nemery**

Belgium

**Yang Bai**

Belgium

**Nicolas Lanthier**

Belgium

**Ghislaine Gayan-Ramirez**

Belgium

**Blanca Himes**

USA

**Bettina F Loza**

Netherlands

**Brian McCullagh**

Ireland

**Eanes Pereira**

Brazil

**Daniela Andaku**

Brazil

**Lia Bittencourt**

Brazil

**Mariana Ferreira**

Brazil

**Andre Albuquerque**

Brazil

**Emilio Pizzichini**

Brazil

**Bruno Bezerril Andrade**

Brazil

**Bruno Balbi**

Italy

**Barbara Yawn**

USA

**Collin Brooks**

New Zealand

**Craig Winstanley**

UK

**Luca Cabrini**

Italy

**Michael Parkins**

Canada

**Bradley Quon**

Canada

**Pat Camp**

Canada

**Michael Stickland**

Canada

**Gambo Aliyu**

Canada

**John Garvey**

Canada

**Dennis Jensen**

Canada

**Jeremy Road**

Canada

**Corey Tomczak**

Canada

**Adam Shapiro**

Canada

**Adrian West**

Canada

**David Ngan**

Canada

**Anthony D'Urzo**

Canada

**Carlos Aravena**

Chile

**Carlo Caffarelli**

Italy

**Carlos Martinez**

USA

**Carsten Schwarz**

Germany

**Paolo Ceppi**

Italy

**Wenjun Mou**

China

**Nong J Zhang**

China

**Duo Zhang**

China

**Mingzhou Guo**

China

**Yan Jia**

China

**Tianwen Lai**

China

**Sun Hongmei**

China

**Eusebi Chiner**

Spain

**Davide Chiumello**

Italy

**Christian Becker**

USA

**Christophe Dooms**

Belgium

**Christophe Miles**

France

**Gianluca Campo**

Italy

**John Cockroft**

UK

**Connie Yang**

Canada

**Sanja Popovic-Grle**

Croatia

**Davor Plavec**

Croatia

**Christina Tischer**

Germany

**Vitezslav Kolek**

Czech Republic

**Olga Krystufkova**

Czech Republic

**Martina Vasakova**

Czech Republic

**Martina Sterclova**

Czech Republic

**Lukas Capek**

Czech Republic

**David Bennett**

Italy

**Deborah Assayag**

Canada

**Runa Vavia Fenger**

Denmark

**Dilip Shah**

USA

**A. Daniel Martin**

USA

**Alarico Ariani**

Italy

**Deepa Rastogi**

USA

**Elżbieta Radzikowska**

Poland

**Edurne Almirall**

Spain

**Elena Gimeno-Santos**

Spain

**Eliecer Coto**

Spain

**Emily Wan**

USA

**Eduard Monsó**

Spain

**Erica Rutten**

Netherlands

**Kelemework Adane**

Ethiopia

**Eric Wong**

Canada

**Fabiano Di Marco**

Italy

**Felip Burgos**

Spain

**Olli Ruuskanen**

Finland

**Francesco Puma**

Italy

**Raphael Borie**

France

**Fanny Rancière**

France

**Amelie Guihot**

France

**Sylvie Boisramé**

France

**Stanislas Ledochowski**

France

**Sebastien Chanoine**

France

**Bernard Maitre**

France

**Bruno Crestani**

France

**Francesco Bonella**

Germany

**Ertunc Altiok**

Germany

**Christian G Cornelissen**

Germany

**Gernot Zissel**

Germany

**Nico Derichs**

Germany

**Matthias Griese**

Germany

**Saeed Kolahian**

Germany

**Joachim Riethmüller**

Germany

**Elaine Fuertes**

Germany

**Melanie Königshoff**

Germany

**Helge Hebestreit**

Germany

**Johannes Bickenbach**

Germany

**Alexander Kersten**

Germany

**Jan Spillner**

Germany

**Nils von Neuhoff**

Germany

**Erich Stoelben**

Germany

**Johanna Luca Leinert**

Germany

**Joachim Müller-Quernheim**

Germany

**Michael Kreuter**

Germany

**Nikolaus Becker**

Germany

**Jan Hendrik Storre**

Germany

**Thomas Karlas**

Germany

**Florian Fuchs**

Germany

**Peter Kardos**

Germany

**Heinrich Worth**

Germany

**Dominik Hartl**

Germany

**Peter Gilligan**

USA

**Giuseppe Verlato**

Italy

**Theodore Dassios**

Greece

**Christina Kalogeropoulou**

Greece

**Sophia Schiza**

Greece

**Izolde Bouloukaki**

Greece

**Spyros Papiris**

Greece

**Eirini Roumpedaki**

Greece

**Giovanni Sotgiu**

Italy

**Nabeel Hamzeh**

USA

**Hans Jörg Baumann**

Germany

**Helena Backman**

Sweden

**Herbert Schiller**

Germany

**Hui Fang Lim**

Singapore

**Veronika Müller**

Hungary

**Jozsef Lovey**

Hungary

**Ian Sayers**

UK

**Magnus Gottfredsson**

Iceland

**Joyashree Banerjee**

India

**Anurag Agrawal**

India

**Arjun Ram**

India

**Susi Ari Kristina**

Indonesia

**Indre Butiene**

Lithuania

**Mehrnoosh Doroudchi**

Iran

**Liam Cormican**

Ireland

**Shane O'Neill**

Ireland

**Limy Wong**

Ireland

**Anne Costello**

Ireland

**Kirsten Schaffer**

Ireland

**Donald Silverberg**

Israel

**Oleg Dolkart**

Israel

**Antonello Nicolini**

Italy

**Davide Colombi**

Italy

**Cristiano Rampinelli**

Italy

**Leonello Fuso**

Italy

**Francesco Pistelli**

Italy

**Claudia Ravaglia**

Italy

**Dina Visca**

Italy

**Giovanni Volpicelli**

Italy

**Fabrizio Luppi**

Italy

**Silvano Gallus**

Italy

**Lara Pisani**

Italy

**Marco Confalonieri**

Italy

**Claudio Pedone**

Italy

**Marco Falcone**

Italy

**Maddalena Giannella**

Italy

**Simone Dore**

Italy

**Cristiano Fava**

Italy

**Claudia Crimi**

Italy

**Monica Rocco**

Italy

**Salvatore Cazzato**

Italy

**Michele Mondoni**

Italy

**Paola De Filippi**

Italy

**Paolo Scanagatta**

Italy

**Mario Olivieri**

Italy

**Giulio Rossi**

Italy

**Annunziata Faustini**

Italy

**Raffaele Antonelli-Incalzi**

Italy

**Alessandro Marcon**

Italy

**Luca Ampollini**

Italy

**Alessio Rungatscher**

Italy

**Cinzia Maspero**

Italy

**Cristina Cigana**

Italy

**Nicola Ciancio**

Italy

**Federico Lavorini**

Italy

**Mario Mascalchi**

Italy

**Annalisa Carlucci**

Italy

**Guido Vagheggini**

Italy

**Francesco Fanfulla**

Italy

**Carmelo Caserta**

Italy

**Mario Cazzola**

Italy

**Susanna Esposito**

Italy

**Iraklis Tsangaris**

Greece

**Isabelle Vivodtzev**

France

**Joanna Chorostowska-Wynimko**

Poland

**Hans Daniels**

Netherlands

**Jacques Pralong**

Swaziland

**Janet Williams**

Australia

**Yoshinobu Eishi**

Japan

**Shinji Sasada**

Japan

**Toru Hifumi**

Japan

**Shinji Abe**

Japan

**Takushi Namba**

Japan

**Takashi Motegi**

Japan

**Akira Koarai**

Japan

**Mizuho Nishio**

Japan

**Masayuki Hojo**

Japan

**Atsushi Sano**

Japan

**Masashi Bando**

Japan

**Masanori Kitaichi**

Japan

**Takeshi Kawasaki**

Japan

**Takeshi Johkoh**

Japan

**Atsushi Nambu**

Japan

**Nobuhiko Nagata**

Japan

**Jeffrey Horowitz**

USA

**Joao Winck**

UK

**Javier Diaz-Mendoza**

USA

**Jean-Francois Mornex**

France

**Jessica Kasza**

Australia

**Jose Herazo-Maya**

USA

**Joseph Keane**

Ireland

**Konstantinos Samitas**

Greece

**Karin Valkenet**

Netherlands

**Barbara Kahl**

Germany

**Kumiko Kanatani**

Japan

**Daniel Kass**

USA

**Karen Bernard**

USA

**Kevin Pethe**

Singapore

**Seong Yong Lim**

South Korea

**Heedoo Lee**

South Korea

**Gi-Byoung Nam**

South Korea

**Stefan Krüger**

UK

**Laura Crotty Alexander**

USA

**Chung-Tat Lun**

Hong Kong

**Eleftherios Zervas**

Greece

**Lenka Munoz**

Australia

**Leslie Grammer**

USA

**Kathleen Lindell**

USA

**Lokesh Guglani**

USA

**Louisa Owens**

Australia

**Martin Kneyber**

Netherlands

**Massimo Corradi**

Italy

**Mark Hew**

Australia

**Michael Marcus**

UK

**Marlies Wijseenbeck**

Netherlands

**Jordan Minov**

Macedonia

**Marcin Zielinski**

Poland

**Mariano Mazzei**

Argentina

**Maria Molina-Molina**

Spain

**Maria Rosaria Bonsignore**

Italy

**Martin Andersson**

Sweden

**Masahide Oki**

Japan

**Stamatoula Tsikrika**

Greece

**Mazen Al-Alawi**

Ireland

**Michael Dreher**

Germany

**Megan Jensen**

Australia

**Jorge Ivan Gamez-Nava**

Mexico

**Luis Teran**

Mexico

**Michael Abramson**

Australia

**Michael Hogardt**

Germany

**Michal Shteinberg**

UK

**Claudio Micheletto**

Italy

**Mark Jennings**

USA

**Mohamed Manji**

Tanzania

**Alberto García-Basteiro**

Mozambique

**Monica Goldklang**

USA

**Nicholas Glanville**

UK

**Noelia Cubero de Frutos**

Spain

**A Knoops**

Netherlands

**Ivo Wiertz**

Netherlands

**Pieter Hiemstra**

Netherlands

**Mark Koelemay**

Netherlands

**Job van der Palen**

Netherlands

**Neuza Silva**

Portugal

**Nicola Murgia**

Sweden

**Nicola Sverzellati**

Italy

**Nicolas Girard**

France

**Nigel Scawn**

UK

**Ademola Fawibe**

Nigeria

**Niranjan Jeganathan**

USA

**Jo Roislien**

Norway

**Martin Gerdes**

Norway

**Monica Linea Vold**

Norway

**Tehmina Mustafa**

Norway

**Inger Schou Bredal**

Norway

**Cecilie Svanes**

Norway

**Ole-Gunnar Anfinsen**

Norway

**Pankaj Bhavsar**

UK

**Nousheen Pradhan**

Pakistan

**Peter Herbison**

New Zealand

**Pierre Goussard**

South Africa

**Brian Pickering**

UK

**Pietro Pirina**

Italy

**Wieslawa Duszynska**

Poland

**Magdalena Muc**

Poland

**Janos Porszasz**

USA

**Tiago Jacinto**

Portugal

**Joao Fonseca**

Portugal

**Yu Li**

China

**Robert Whiley**

UK

**Rajesh Bhagat**

USA

**Rachel Evans**

UK

**Roberto Santa Cruz**

UK

**Ritesh Agarwal**

India

**Ryang Hwa Lee**

USA

**Rajiv Machado**

UK

**Robert Siebers**

New Zealand

**Kari Roberts**

UK

**Roland Leung**

Hong Kong

**Stephen Ruoss**

USA

**Vsevolod Kuzkov**

Russia

**Shawn Aaron**

Canada

**Samantha Aso**

Spain

**Mohamed Regal**

Saudi Arabia

**Sanjay Chotirmall**

Singapore

**Souheil El-Chemaly**

USA

**Sergey Dikalov**

USA

**Sergio Campainha**

Portugal

**Szymon Skoczynski**

Poland

**Yick Hou Lim**

Singapore

**David Price**

Singapore

**Sanjay Haresh Chotirmall**

Singapore

**Simon Walsh**

UK

**Simon Podnar**

Slovenia

**Ivan Solovic**

Slovakia

**Greg Calligaro**

South Africa

**Keertan Dheda**

South Africa

**Felipe Cardenal**

Spain

**Ricard Ramos**

Spain

**Antonio Esquinas**

Spain

**Maria Jesus Garcia**

Spain

**Ernest Nadal**

Spain

**Jose L Vercher Conejero**

Spain

**Catia Cilloniz**

Spain

**Irene Latorre**

Spain

**Esther Garcia Gil**

Spain

**Salut Santos**

Spain

**Stefano Baglioni**

Italy

**Stefano Sestini**

Italy

**Stylianos Vittorakis**

Greece

**Stephan Rosenkranz**

Germany

**Stephanie Christenson**

USA

**Stephen Thielke**

USA

**Sureshbabu Angara**

USA

**Ann Lindberg**

Sweden

**Cecilia Stålsby Lundborg**

Sweden

**Linnea Hedman**

Sweden

**Jenny Hallberg**

Sweden

**Konrad Bloch**

Switzerland

**Wolfgang Jungraithmayr**

Switzerland

**Werner C Albrich**

Switzerland

**Sebastian R Ott**

Switzerland

**Suzanne Cloonan**

USA

**Toshiaki Jibiki**

UK

**Tomas Carroll**

Ireland

**Teck Boon Low**

Singapore

**Pongdhep Theerawit**

Thailand

**Rabindra Tirouvanziam**

USA

**Terumi Midoro-Horiuti**

USA

**Tony Velkov**

Australia

**Toshinori Takada**

Japan

**Guniz Meyanci Koksal**

Turkey

**Baykal Tulek**

Turkey

**Hasan Bayram**

Turkey

**Deus Lukoye**

Uganda

**Rashid Nadeem**

United Arab Emirates

**Iram Haq**

UK

**Kenneth Macleod**

UK

**Sally Singh**

UK

**Andrew Wilson**

UK

**Adrian Martineau**

UK

**Jennifer Quint**

UK

**Shoaib Faruqi**

UK

**Ronan Breen**

UK

**Timothy Hinks**

UK

**Daniel Bratton**

UK

**Helen Chapel**

UK

**Dhruv Parekh**

UK

**Ghazwan Butrous**

UK

**Peter Callery**

UK

**Andrew Fall**

UK

**David Spencer**

UK

**Rachel Jordan**

UK

**Andrew Bush**

UK

**Michael Hughes**

UK

**Prasad Nagakumar**

UK

**Xabier Cortés-Lavaud**

UK

**Francis Gilchrist**

UK

**Simon Langton Hewer**

UK

**Roma Maguire**

UK

**Thomas Wiliams**

UK

**Eduardo Fernandez Moya**

UK

**Claire Hogg**

UK

**Sooky Lum**

UK

**Neil Thomson**

UK

**Clive Page**

UK

**Jonathan Cohen**

UK

**Gary Latchford**

UK

**Jens Madsen**

UK

**Robert Guzy**

USA

**Julie Ledford**

USA

**Douglas Curran-Everett**

USA

**Cory Hogaboam**

USA

**Jiwang Chen**

USA

**Haiyang Tang**

USA

**Pui-Ying Iroh Tam**

USA

**Stacey Martiniano**

USA

**Anurag Bajaj**

USA

**Carolyn Calfee**

USA

**Stephen Chan**

USA

**Ying Sun**

USA

**Peter Moschovis**

USA

**Hitendra Chand**

USA

**Venkateshwar Mutyam**

USA

**Maor Sauler**

USA

**Girish Nair**

USA

**Jose Gomez**

USA

**Rabindra Tirouvanziam**

USA

**Natasha Williams**

USA

**Forest Arnold**

USA

**Deepa Rastogi**

USA

**Kameswara Rao Badri**

USA

**Dong-Mei Wang**

USA

**Jonathan Kropski**

USA

**Surya Bhatt**

USA

**Raksha Jain**

USA

**Deog Kyeom Kim**

USA

**Bo Shen**

USA

**Michael Gentile**

USA

**Samir Kelada**

USA

**Emily Wan**

USA

**Khalil Bdeir**

USA

**Brian Graham**

USA

**Talat Islam**

USA

**Jules Lin**

USA

**Stephen Aronoff**

USA

**John Ferguson**

USA

**Rama Mallampalli**

USA

**Neil Alexis**

USA

**Amit Gaggar**

USA

**Timothy Weaver**

USA

**William Gerthoffer**

USA

**Matthew Foster**

USA

**Steve Georas**

USA

**Karen Bernard**

USA

**Louise Hecker**

USA

**Behrouz Jafari**

USA

**Jessica Bon**

USA

**Yashoda Hosakote**

USA

**Tejaswini Kulkarni**

USA

**Sachin Gupte**

USA

**Jennifer Trittmann**

USA

**Suzanne M Cloonan**

USA

**Praveen Mannam**

USA

**Edward Miranda**

USA

**Minfeng Shu**

USA

**Chao-Hung Lee**

USA

**Anna Kurdowska**

USA

**Angela Rogers**

USA

**Carol Conrad**

USA

**Ashish Sharma**

USA

**Asli Gorek Dilektasli**

USA

**Anand Iyer**

USA

**Mehdi Mirsaeidi**

USA

**Kenneth Berger**

USA

**Janos Porszasz**

USA

**Trisha Parekh**

USA

**Hernando Gomez**

USA

**Philip Westgate**

USA

**Gary M Hunninghake**

USA

**Imre Noth**

USA

**Benjamin Kopp**

USA

**Nektarios Barabutis**

USA

**Ming-Hui Fan**

USA

**Cherry Wongtrakool**

USA

**Vineet Bhandari**

USA

**Joseph Swanson**

USA

**Harikrishnan Parameswaran**

USA

**Parag Desai**

USA

**Barry Shea**

USA

**Vanessa Garcia-Larsen**

UK

**Victor Kim**

USA

**Venkateshwar Mutyam**

USA

**Dong-Mei Wang**

USA

**Xavier Solanich**

Spain

**Xue Li Guan**

Singapore

**Zachary Sellers**

USA

**Zsolt Pápai-Szekely**

Hungary

**Zhiyu Dai**

USA

